# Evolutionary Trends and Research Focal Points on Port Wine Stains: A Scientometric and Meta‐Analysis

**DOI:** 10.1111/jocd.16770

**Published:** 2025-02-07

**Authors:** Zheren Su, Xuanfeng Chen, Rui Zhang, Jing Li, Zifu Zhou, Jianhai Bi, Ran Huo

**Affiliations:** ^1^ Department of Plastic and Aesthetic Surgery, Shandong Provincial Hospital, Cheeloo College of Medicine Shandong University Jinan China; ^2^ Department of Plastic Surgery The First Affiliated Hospital of Fujian Medical University Fuzhou China; ^3^ Department of Plastic and Aesthetic Surgery Shandong Provincial Hospital Affiliated to Shandong First Medical University Jinan China

**Keywords:** bibliometrics, meta‐analysis, port wine birthmarks, vascular anomalies

## Abstract

**Background:**

Despite numerous studies over the past two decades, clinical treatment for port wine stains (PWS) has shown limited progress. Analyzing evolutionary trends and research focal points can illuminate current deficiencies and guide future investigations. We aim to conduct a scientometric analysis and meta‐analysis to uncover the historical trajectory, research hotspots, and future directions of PWS.

**Methods:**

We conducted a scientometric analysis of articles related to PWS published between 2000 and 2023. Data were retrieved from the Web of Science Core Collection (WoSCC) and analyzed using R software, VOSviewer, and CiteSpace. Furthermore, we retrieved articles reporting therapeutic clearance rates for PWS from PubMed, Embase, Web of Science, and Cochrane search engines. A meta‐analysis was performed using Stata/MP to assess whether treatment outcomes for PWS have improved in the 21st century.

**Results:**

The annual scientific output was stable from 2000 to 2023. The top three countries in terms of document production were the United States, China, and the United Kingdom. Pulsed dye laser (PDL) and photodynamic therapy (PDT) were the two primary modalities used in the treatment of PWS. A notable difference exists in the preference for these treatment modalities between China and Western countries. The meta‐analysis shows improvement in treatment outcomes for PWS from 2000 to 2023.

**Conclusions:**

Although limited, treatment outcomes for PWS have shown improvement in the 21st century. However, there is a critical need for research directions that could revolutionize current treatment practices. Genetic discoveries have suggested promising therapeutic targets for potential breakthroughs in the future.

## 1 Introduction

Port wine stains (PWS) are the most common congenital vascular malformations, affecting approximately 0.3% of children [1, 2]. These birthmarks are characterized by ectatic capillaries and postcapillary venules located in the papillary and mid‐reticular layers of the dermis [3]. PWS typically appear as erythematous patches initially, progressively ectatic and eventually developing nodularity or hypertrophy in approximately two thirds of patients by the fifth decade of life [4]. The aberrant cosmetic appearance due to their frequent occurrence on the head and neck, coupled with a tendency for spontaneous bleeding, significantly impacts both the physical and psychological well‐being of patients [5].

Despite numerous studies over the past two decades, clinical treatment outcomes for PWS have shown limited progress. Analyzing evolutionary trends and research focal points can illuminate current deficiencies and guide future investigations. Bibliometric analysis, known as scientometric analysis in scientific fields, employs quantitative and statistical methods to visualize evidence based on published research articles [6, 7]. Therefore, we aimed to conduct a scientometric study focusing on literature published from 2000 to 2023. Furthermore, to address whether treatment outcomes for PWS have improved, we performed a meta‐analysis integrating existing evidence on clearance rates for PWS.

## 2 Methods

### 2.1 Data Source and Search Strategy for Scientometric Analysis

**FIGURE 1:**
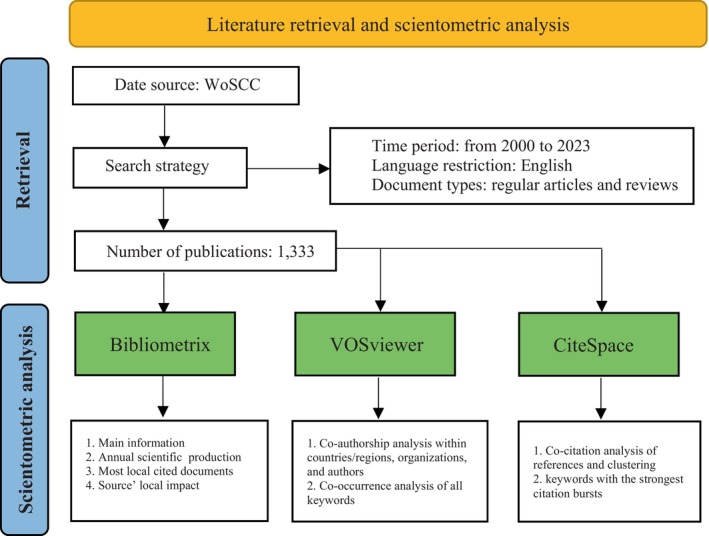
Flowchart illustrating the scientometric analysis process.

The Web of Science Core Collection (WoSCC) was selected for its extensive global academic coverage. We conducted literature retrieval on January 11, 2024, using the following search strategy: “port wine stains*” (Topic) OR “n$evus flammeus” (Topic). The search was confined to articles published between 2000 and 2023, encompassing regular articles and reviews, and restricted to English‐language publications. To analyze research trends and hotspots in PWS, we used “port wine stains” and “nevus flammeus” as primary keywords and restricted the search to the Topic field, encompassing titles, abstracts, and keywords, to ensure relevance. The entire process of literature retrieval and scientometric analysis is summarized in Figure 1.

### 2.2 Software for Scientometric Analysis

Scientometric analysis was conducted using several tools: the Bibliometrix R package (version 4.1.4) within R software (version 4.2.3), VOSviewer (version 1.6.20), and CiteSpace (version 6.3.1) [8, 9, 10]. GraphPad Prism 10 (version 10.2.0) and SCImago Graphica (version 1.0.42) were utilized for visualizing specific results from these tools.

Bibliometrix R Package: Utilized to provide a comprehensive overview of scientific production on PWS. Specifically, we utilized the “Main Information” and “Annual Scientific Production” modules to obtain the distribution of document types and track the annual publication trends. The “Most Local Cited Documents” and “Sources' Local Impact” modules helped identify the top 10 journals and articles with the highest impact.

VOSviewer: Used for visualizing collaborative networks within authors, organizations, and countries through co‐authorship analysis. We also conducted a co‐occurrence analysis of all keywords, including author keywords and keywords plus.

CiteSpace: Employed to visualize the co‐citation network of references and identify clusters. Additionally, we generated the top 20 keywords with the strongest citation bursts.

### 2.3 Literature Inclusion and Risk of Bias Assessment for Meta‐Analysis

Published studies reporting therapeutic clearance rates for PWS were systematically identified using PubMed, Embase, Web of Science, and Cochrane search engines on April 16, 2024. The search strategy employed in PubMed was as follows: ((“nevus flammeus” [Title/Abstract]) OR (“naevus flammeus” [Title/Abstract]) OR (“port wine stains*” [Title/Abstract]) OR (Port‐Wine stains [MeSH Terms])) AND ((laser [Title/Abstract]) OR (photodynamic [Title/Abstract]) OR (lasers [MeSH Terms])). The publication period was restricted to articles published from 2000 to 2023, and only regular articles written in English were considered.

Retrieved articles were deduplicated and screened using EndNote X9. We included studies that reported therapeutic clearance rates categorized into quartiles of lighting percentage (0%–25%, 26%–50%, 51%–75%, 76%–100%) or other percentage ranges that could be converted. The quality of the included articles was assessed using the Grading of Recommendations Assessment, Development and Evaluation (GRADE) approach [11].

### 2.4 Meta‐Analysis

Treatment modalities and clearance rates were extracted. Clearance rates above 50% were selected as the outcome indicator for aggregating the extracted data. Subsequently, a meta‐analysis, including subgroup analyses, was performed in Stata/MP (version 15.1) using the command: metaprop num denom, random by (subgroup) ftt cimethod (score) label (namevar = study). The meta‐analysis utilized the Freeman‐Tukey double arcsine (ftt) method and cimethod (score) approach within the metaprop module, specifically designed for analyzing proportions in Stata software [12]. Here, the numerator represents the number of cases with clearance rates above 50%, and the denominator denotes the total number of cases.

## 3 Results

### 3.1 Global Publication Trends

**FIGURE 2:**
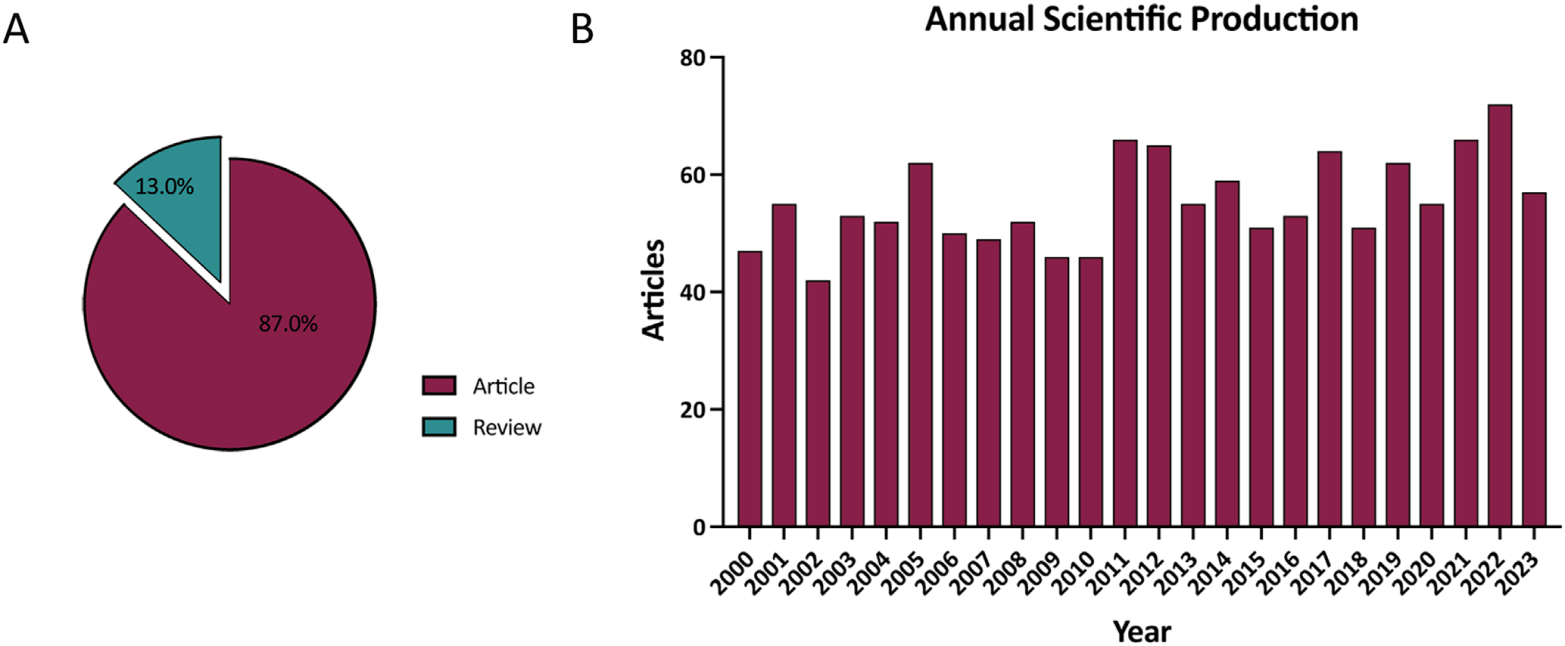
Scientific production on port wine stains (PWS). (A) Distribution of document types. (B) Annual production from 2000 to 2023.

A total of 1333 articles were included for scientometric analysis, consisting of 87.0% regular articles and 13.0% review articles (Figure 2A). The annual scientific production remained relatively stable, ranging between 50 and 60 articles in most years from 2000 to 2023 (Figure 2B).

### 3.2 Journals and Articles

Table 1 lists the top 10 journals with the highest h‐index on PWS from 2000 to 2023, where the h‐index is defined as the number of papers with citations equal to or exceeding “h” [13]. A total of 477 articles were published across these 10 journals, which accounted for approximately one‐third of the retrieved articles. Table 2 displays the top 10 local cited articles, determined by their citation counts among the retrieved articles.

**TABLE 1:** The top 10 journals with the highest h‐index on port wine stains (PWS) from 2000 to 2023.

Journal	H‐index	Number of publications	Impact factor (2023)	JCR quartile (2023)
LASERS IN SURGERY AND MEDICINE	42	139	2.2	Q2
DERMATOLOGIC SURGERY	31	71	2.5	Q1
JOURNAL OF THE AMERICAN ACADEMY OF DERMATOLOGY	28	46	12.8	Q1
BRITISH JOURNAL OF DERMATOLOGY	25	36	11.0	Q1
PHOTODIAGNOSIS AND PHOTODYNAMIC THERAPY	17	44	3.1	Q2
JOURNAL OF BIOMEDICAL OPTICS	16	29	3.0	Q2
LASERS IN MEDICAL SCIENCE	16	65	2.1	Q2
JAMA DERMATOLOGY/ARCHIVES OF DERMATOLOGY	14	15	11.5	Q1
PHYSICS IN MEDICINE AND BIOLOGY	13	15	3.3	Q1
AMERICAN JOURNAL OF MEDICAL GENETICS PART A	11	17	1.7	Q3

**TABLE 2:** The top 10 local cited articles on port wine stains (PWS) from 2000 to 2023.

Article title	Journal	Year	Local citations
An overview of clinical and experimental treatment modalities for port wine stains	J AM ACAD DERMATOL	2012	96
Treatment of pulsed dye laser‐resistant port wine stain birthmarks	J AM ACAD DERMATOL	2007	87
Long‐pulsed neodymium: yttrium‐aluminum‐garnet laser treatment for port wine stains	J AM ACAD DERMATOL	2005	85
Capillary malformation‐arteriovenous malformation, a new clinical and genetic disorder caused by RASA1 mutations	AM J HUM GENET	2003	51
Treatment of port wine stain birthmarks using the 1.5‐msec pulsed dye laser at high fluences in conjunction with cryogen spray cooling	DERMATOL SURG	2002	47
Hypertrophy in port wine stains: prevalence and patient characteristics in a large patient cohort	J AM ACAD DERMATOL	2012	46
Combined 595‐nm and 1064‐nm laser irradiation of recalcitrant and hypertrophic port wine stains in children and adults	DERMATOL SURG	2009	45
Confocal microscopy study of nerves and blood vessels in untreated and treated port wine stains: preliminary observations	DERMATOL SURG	2004	44
Quality of life in adults with facial port wine stains	J AM ACAD DERMATOL	2017	44
Thickening and nodules in port wine stains	J AM ACAD DERMATOL	2001	43

### 3.3 Countries and Regions

**FIGURE 3:**
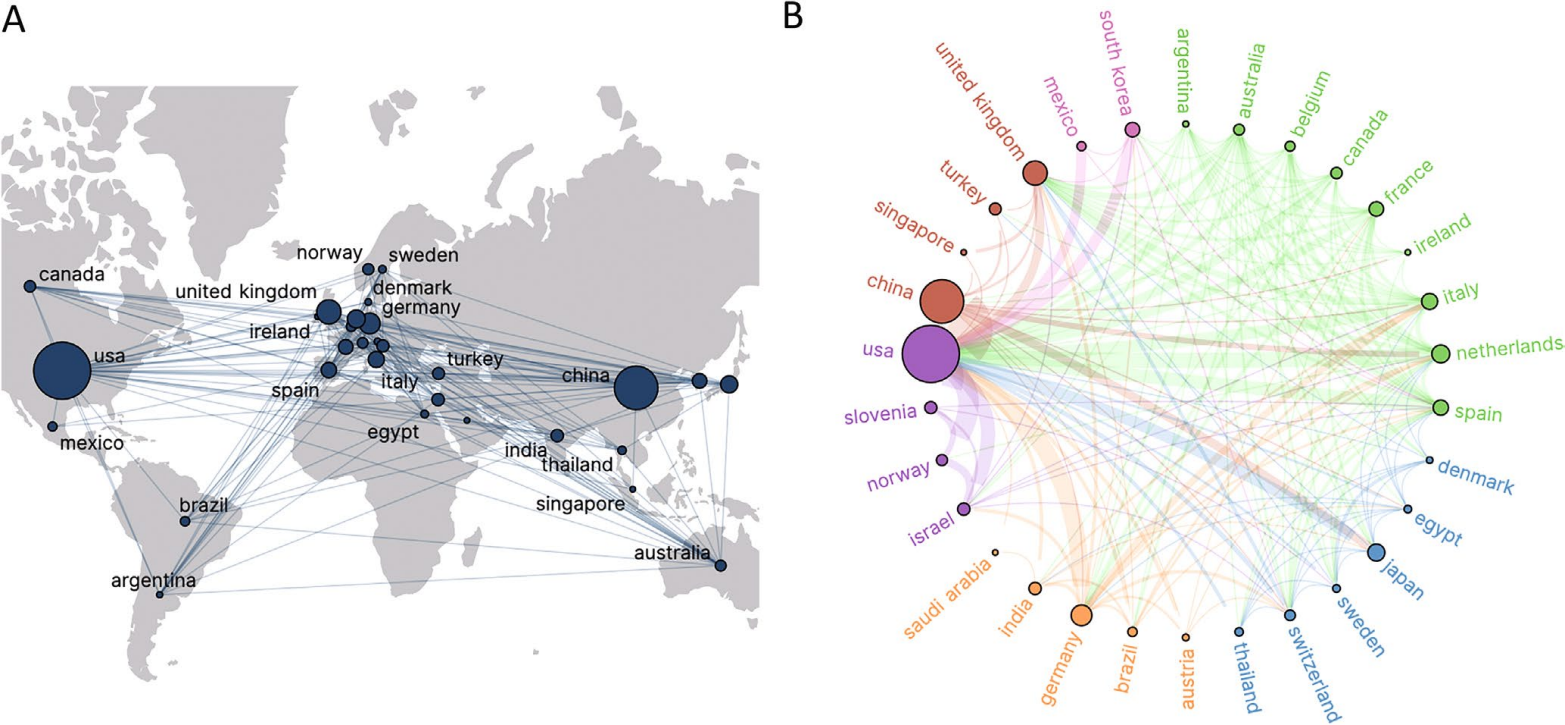
Distribution of countries/regions. (A) Geographic distribution of collaboration between countries/regions (Top 30). (B) Network visualization of collaboration between countries/regions (Top 30).

**FIGURE 4:**
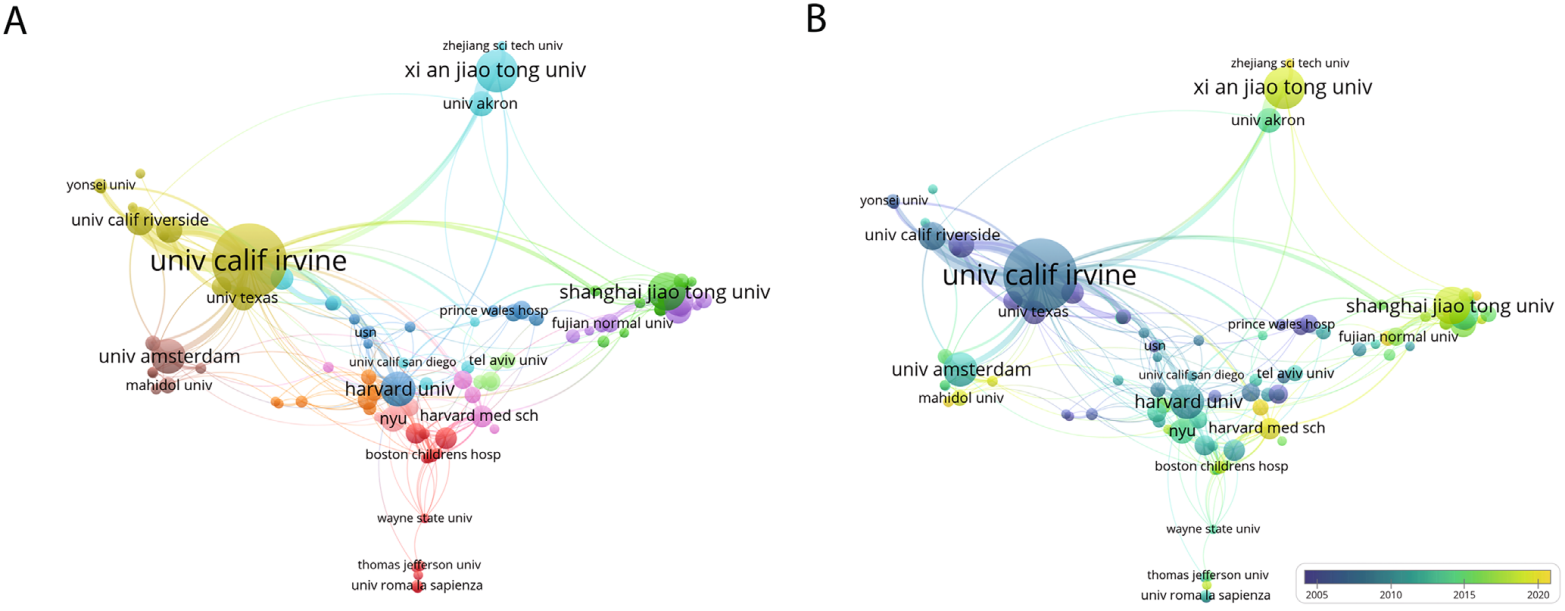
Distribution of universities/institutions. (A) Network visualization of collaboration between universities/institutions (Top 100). (B) Overlay visualization of collaboration between universities/institutions (Top 100).

**FIGURE 5:**
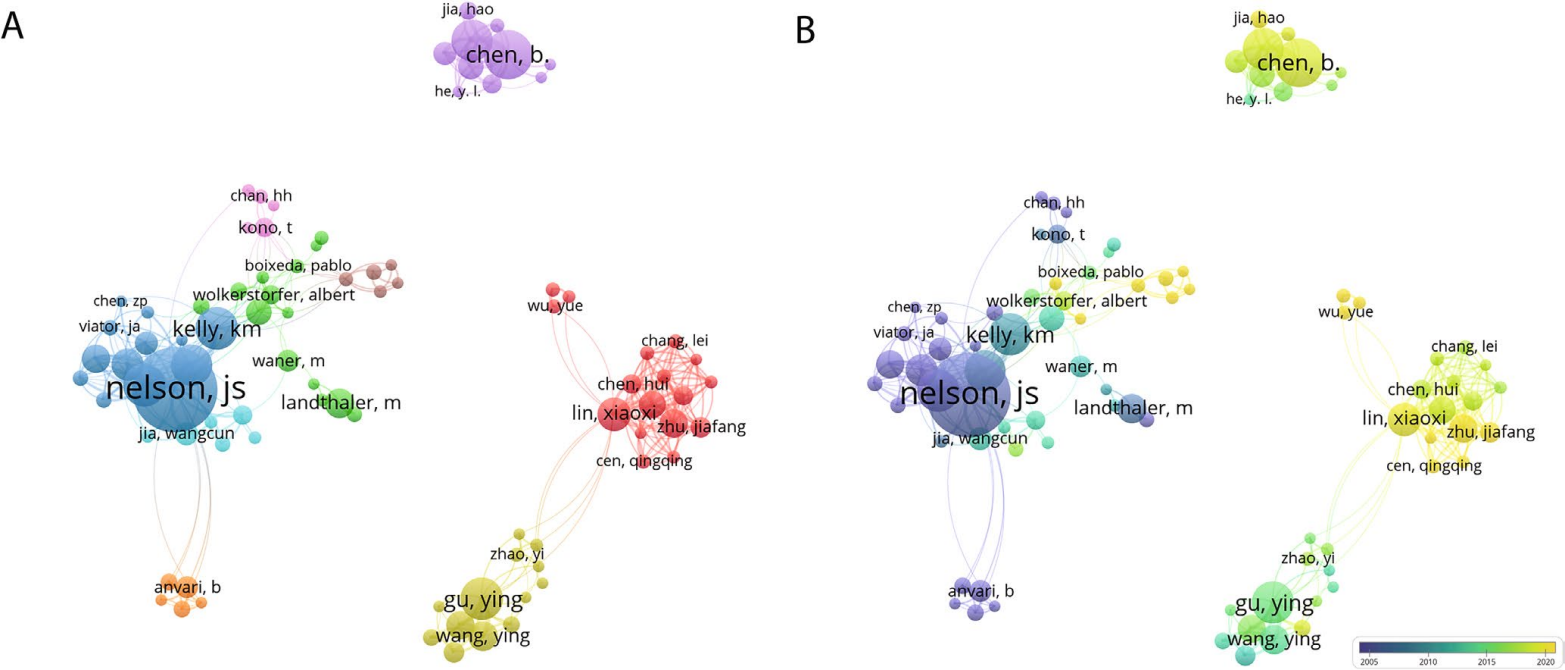
Distribution of authors. (A) Network visualization of collaboration between authors (Top 100). (B) Overlay visualization of collaboration between authors (Top 100).

In total, 62 countries/regions contributed to documents on PWS, with the top 30 selected for further investigation. Figure 3 depict this distribution, where node sizes correspond to document volume and links between nodes indicate collaboration strength, like Figure 4 and Figure 5 presented subsequently. Figure 3B shows six clusters, each represented by a different color, illustrating close connections among countries/regions, akin to the representations in Figures 4A and 5A. The top three countries/regions in terms of document production were the United States (555/1333, 41.6%), China (322/1333, 24.2%), and the United Kingdom (99/1333, 7.4%).

### 3.4 Universities and Institutions

A total of 1450 organizations contributed documents on PWS, with the top 100 organizations selected for subsequent analysis. As shown in Figure 4A, the top three organizations in terms of document production were the University of California, Irvine (133/1333, 10.0%), Xi'an Jiaotong University (52/1333, 3.9%), and Shanghai Jiao Tong University (43/1333, 3.2%). As shown in Figure 4B, node colors denote the average publication year, reflecting the collective publication year average of all documents within a literature collection, akin to Figure 5B. The University of California, Irvine has been pioneering in this field, while Xi'an Jiaotong University has made notably contributions in recent years.

### 3.5 Authors

A total of 4725 authors contributed to documents on PWS, with the top 100 authors selected for detailed analysis. Lin Xiaoxi, Albert Wolkerstorfer, J. Stuart Nelson were the authors who had the strongest collaborative relationships within the top three biggest clusters (Figure 5A). J. Stuart Nelson, who pioneered earlier in the field, had the largest publication volume and the highest number of citations (Figure 5B). Notably, several of these clusters predominantly comprised Chinese authors.

### 3.6 Keywords and Trend Topics

**FIGURE 6:**
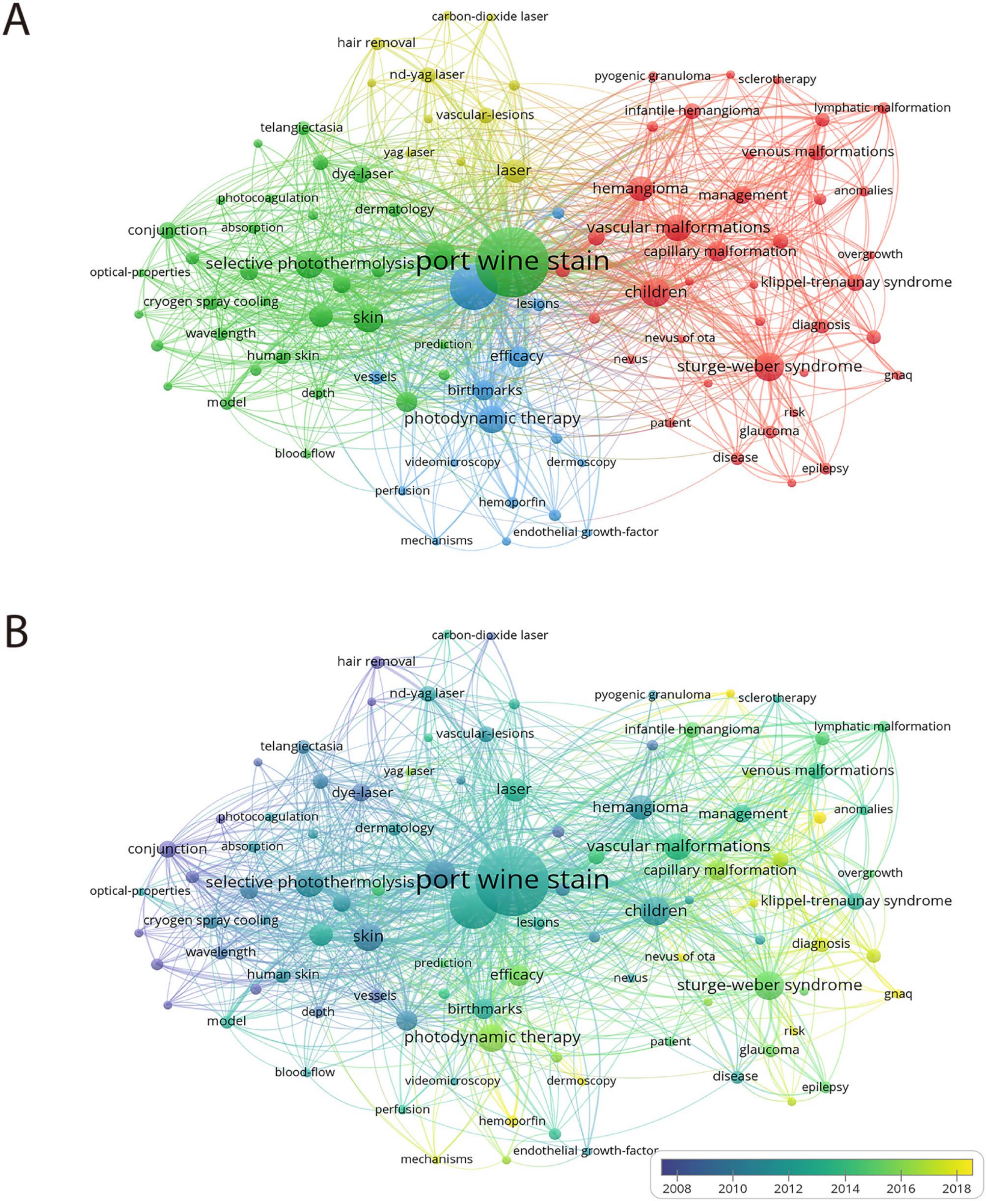
Distribution of keywords. (A) Network visualization of keywords (Top 100). (B) Overlay visualization of keywords (Top 100).

**FIGURE 7:**
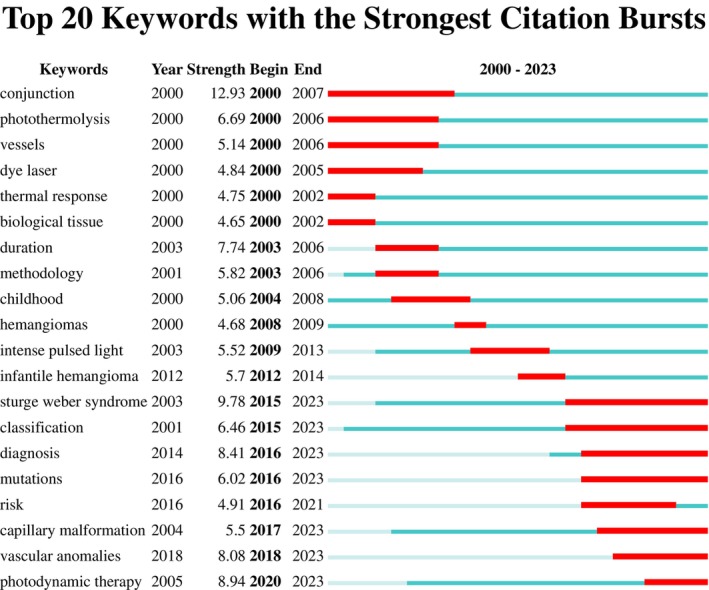
Top 20 keywords with the strongest citation bursts on port wine stains (PWS) from 2000 to 2023.

A total of 3753 keywords were extracted, with the top 100 keywords selected for further analysis. The parameter settings align with the co‐authorship analysis. Four clusters were identified (Figure 6A). The green cluster predominantly focuses on pulsed dye laser (PDL) and selective photothermolysis. The yellow cluster mainly discusses other laser treatments. The blue cluster is primarily centered around photodynamic therapy (PDT) and Hemoporfin. The red cluster primarily addresses syndromes or diseases with PWS cutaneous manifestations or conditions requiring differential diagnosis. Figure 6B highlights PDT and Hemoporfin as emerging hotspots. Particularly noteworthy is the rise of GNAQ gene as a new research focal point in recent years. To deepen our comprehension of the evolutionary trends, we developed a keyword burst map. As illustrated in Figure 7, PDL experienced a burst of interest from 2000 to 2005, while PDT exhibited a burst from 2020 to 2023.

### 3.7 Co‐Citation References

**FIGURE 8:**
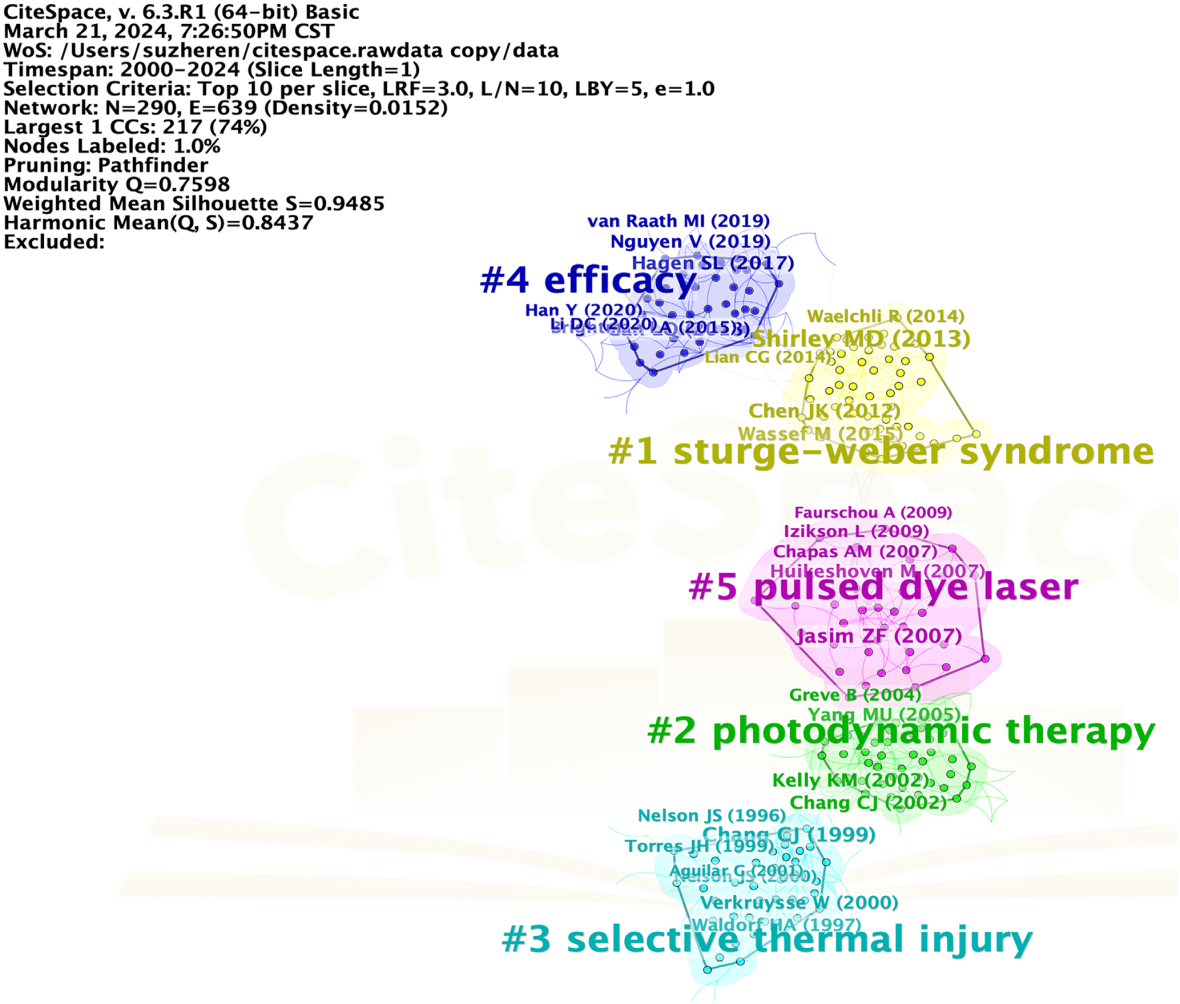
The co‐citation network of references.

We used CiteSpace to visualize the co‐citation network of references, identifying five clusters with significant modularity and high silhouette scores (*Q* = 0.7598, *S* = 0.9485). The network highlights five research focuses: Sturge–Weber syndrome, PDT, selective thermal injury, efficacy, and PDL (Figure 8).

### 3.8 Characteristics of Included Studies for Meta‐Analysis

A total of 2193 records were identified. After removing duplicates and screening, 73 articles were included [14, 15, 16, 17, 18, 19, 20, 21, 22, 23, 24, 25, 26, 27, 28, 29, 30, 31, 32, 33, 34, 35, 36, 37, 38, 39, 40, 41, 42, 43, 44, 45, 46, 47, 48, 49, 50, 51, 52, 53, 54, 55, 56, 57, 58, 59, 60, 61, 62, 63, 64, 65, 66, 67, 68, 69, 70, 71, 72, 73, 74, 75, 76, 77, 78, 79, 80, 81, 82, 83, 84, 85, 86]. The studies primarily focused on PDL and PDT, while other therapies such as neodymium: yttrium‐aluminum‐garnet (Nd:YAG) laser and intense pulsed light (IPL) were also documented. Most of the included studies are observational studies, resulting in the quality of evidence being classified as low or very low.

### 3.9 Results of Meta‐Analysis

**FIGURE 9:**
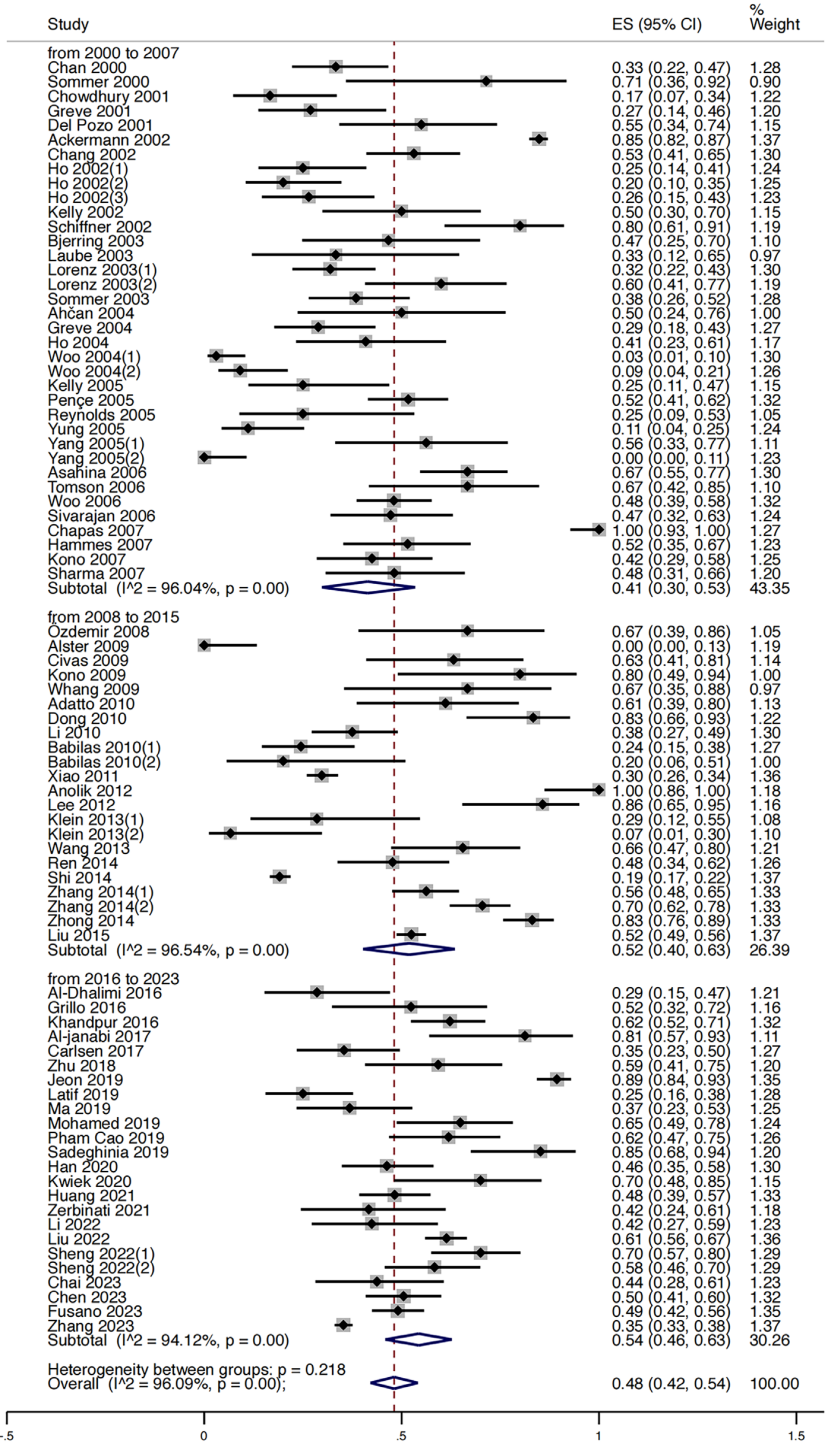
Forest plot of clearance rates reported in port wine stains (PWS) studies published from 2000 to 2023. Effect sizes (ES), representing the proportion of patients achieving ≥ 50% clearance, are presented with 95% confidence intervals (CI).

We conducted a meta‐analysis on the clearance rates of 73 included articles. These articles were divided into three subgroups based on publication years. The pooled estimate of the proportion of patients achieving ≥ 50% clearance was 0.41 (95% CI 0.30–0.53) from 2000 to 2007, 0.52 (95% CI 0.40–0.63) from 2008 to 2015, and 0.54 (95% CI 0.46–0.63) from 2016 to 2023 (Figure 9). The *I* ^2^ of all subgroups was above 90%.

## 4 Discussion

### 4.1 Principal Results

In this study, we conducted a scientometric analysis to summarize global publication information, research collaborations, and research focal points in the field of PWS from 2000 to 2023.

Over the past two decades, the annual scientific output has consistently ranged between 50 and 60 articles per year, reflecting sustained research interest. However, this production volume has not seen significant growth. One notable observation is the absence of major breakthroughs during this period, which has led to a perceived stagnation in research direction and primary issues. This stagnation may be attributed to a preference among researchers for refining existing energy‐based therapies rather than pursuing potentially transformative research directions that could revolutionize current treatment practices.

Furthermore, we have provided a summary of the top 10 journals and articles with the highest impact in the field of PWS. This information aims to facilitate a rapid understanding of key research in the field.

When analyzing collaboration between countries and regions, we identified the United States, China, and the United Kingdom as the top three producers of documents in the field. A notable observation from our author collaboration analysis revealed several clusters predominantly composed of Chinese authors, indicating limited collaboration between China and Western countries. Although some level of cooperation was evident in our analysis of country collaborations, the extent of collaboration appeared to be limited.

Subsequently, we conducted an analysis of keywords to uncover the primary research hotspots. Over the past two decades, PDL has garnered significant attention from researchers, and more recently, PDT has also gained considerable interest. Research has also delved into syndromes or diseases with PWS cutaneous manifestations, including Sturge–Weber syndrome, Klippel‐Trenaunay syndrome, and capillary malformation‐arteriovenous malformation (CM‐AVM). Beyond clinical studies, investigations into the mechanisms of PWS have also emerged, with notable attention in recent years focused on GNAQ gene.

We also conducted an analysis of references, identifying five distinct clusters. PDL and PDT emerged, underscoring their significance in the field of PWS. Furthermore, the “efficacy” cluster reflects researchers' concentrated efforts on improving treatment outcomes for PWS.

Ultimately, we included 73 articles covering various treatment modalities and conducted a meta‐analysis on the clearance rates. Despite numerous studies over the past two decades, clinical treatment outcomes for PWS have shown limited progress. Building on our previous discussion, assessing advancements in PWS treatment could provide insights into whether further efforts to enhance energy‐based therapies are worthwhile, or if exploring research directions that could revolutionize treatment practices would be more pertinent.

A study published in 2019 by van Raath et al. [87] asserted that treatment outcomes for PWS have not improved over the past three decades. They conducted a PubMed search and included studies reporting quartile percentages of clearance rates. Subsequently, they calculated and plotted both the mean clearance per study and a five‐study simple moving average for mean clearance. Upon visual inspection, the findings indicated no upward trend over time. Hence, they concluded that treatment outcomes have not improved. However, their conclusion relied on subjective inspection and evaluation. Furthermore, despite their attempt to mitigate volatility from incidental outliers by plotting five‐study simple moving average, the method likely did not achieve the desired effect due to the narrow time span and inadequate number of studies at each data point. We believe that a meta‐analysis is a more objective and suitable approach to investigate this issue. Thus, we conducted a meta‐analysis and divided the included articles into three subgroups based on publication years: 2000 to 2007, 2008 to 2015, 2016 to 2023. Although no statistical differences were observed, an upward trend in the proportion of patients achieving ≥ 50% clearance was noted across the three subgroups, particularly in the first two subgroups. However, the *I* ^2^ of all subgroups was above 90%, indicating significant heterogeneity within each subgroup, which could potentially impact the credibility of the results.

In our opinion, although limited, treatment outcomes for PWS have shown improvement in the 21st century. This improvement can possibly be attributed to innovative treatment approaches and refined selection of target population. Notably, all 10 studies employing PDT among the 73 included articles were conducted by Chinese researchers [77, 78, 79, 80, 81, 82, 83, 84, 85, 86]. This highlights a discernible difference in treatment modality preference between China and Western countries. Chinese clinicians often favor PDT, especially for treating large PWS, aiming for more uniform fading of lesions. This preference prompts us to question whether these variations arise from differing treatment outcomes influenced by ethnic and Fitzpatrick skin type differences, or if they are simply due to a lack of consensus possibly stemming from insufficient collaborative research efforts.

### 4.2 Implications for Clinical Practice

Although existing treatments have improved, they have not led to significant breakthroughs, highlighting the need for clinicians to explore broader treatment options and engage in further research. Additionally, limited collaboration between Chinese and Western researchers, combined with regional differences in treatment preferences–particularly between PDL and PDT–underscores the importance of international collaboration to harmonize treatment approaches. As treatment modalities continue to diversify, clinicians should consider tailoring their strategies based on individual patient factors, such as age, skin type, and lesion thickness.

### 4.3 Limitations

The exclusive use of WoSCC and the absence of a formal screening process may have resulted in the omission of relevant studies and the inclusion of unrelated ones. Additionally, given the prevalence of citation bias, using citation count as an indicator to identify high‐quality articles may overlook some genuinely valuable literature. Furthermore, the settings of parameters in the analysis, such as thresholds and algorithms, could influence the resulting visualization maps, potentially altering the representation of trends and relationships.

In the meta‐analysis, one limitation is the low quality of the included studies. Additionally, the high *I* ^2^ values in all subgroups indicate significant heterogeneity. Factors such as patient age, lesion thickness, and prior treatment resistance may influence treatment outcomes. Although the relatively large number of studies in each subgroup helps to mitigate distribution imbalances, the uneven distribution of these factors could still act as confounding variables and affect the interpretation of the efficacy results.

### 4.4 Recommendations for Future Work

#### 4.4.1 Early Intervention and Shortened Treatment Intervals With PDL

Since the US Food and Drug Administration (FDA) issued a warning regarding neurodevelopmental risks associated with repeated exposure to general anesthetic and sedation drugs in children younger than 3 years, there has been increased emphasis on initiating treatment for PWS at an earlier age, prioritizing both safety and efficacy [88]. In a retrospective study published by Jeon et al. [68] in 2019, it was found that two‐thirds of patients at the age of 1 year or younger achieved clearance rates ranging from 76% and 100% using PDL, all without the need for general anesthesia. However, Haydar Gray and Holman raised concernes regarding Jeon et al.'s assertion, citing the lack of carefully collected safety data [89]. Mathes and Frieden [90] commented that the high clearance rates might be an overestimation resulted from the physiologic decrease in hemoglobin concentrations. These discussions underscore the necessity for additional clinical evidence to establish the safety and effectiveness of PDL treatment for infants with PWS without the use of general anesthesia. From our perspective, the challenge in accurately performing a differential diagnosis distinguishing between PWS and nevus simplex could potentially contribute to the high clearance rates observed in infants. Furthermore, we are curious about whether the physiologic decrease in hemoglobin concentrations in infants affects the treatment outcomes, considering that hemoglobin acts as the chromophore targeted by PDL.

Additionally, multiple studies have highlighted that early intervention can lead to superior treatment outcomes and alleviate psychological burdens for patients and their families [41, 91, 92, 93, 94, 95, 96, 97]. In 2024, Bajaj et al. [98] published a case series involving 10 patients with a median age of 4 weeks, demonstrating that weekly PDL treatment of PWS can achieve near total or total clearance rates during early infancy. While some research has supported the notion that shorter treatment intervals contribute to enhanced outcomes [38, 54], other studies have reported comparable results across different treatment intervals [67, 99]. Hence, further investigation is warranted to ascertain the optimal treatment intervals for patients of varying ages and Fitzpatrick skin types.

#### 4.4.2 Defining Resistance in PDL Treatment for PWS

While PDL is widely recognized as the gold standard treatment for PWS, some patients may show resistance. When limited efficacy is observed despite multiple PDL treatments, alternative options should be considered. Presently, there still lacks a clear definition of resistant and recalcitrant PWS. Establishing a precise definition is crucial for selecting the target population, promptly adjusting treatment modalities, and avoiding unnecessary treatments. In previous studies, a simple definition characterized resistance as the absence of further improvement or reaching a plateau [63, 100, 101]. A more specific definition specifies resistance as having at least five treatments of PWS but achieved no improvement after the last one or two sessions [80, 102, 103].

These definitions do not encompass cases where significant clearance is achieved, but the lesion remains visibly noticeable, typically due to uneven lightening of PWS lesions. In such cases, further studies are warranted to determine whether alternative treatments, such as PDT, which has been proven effective for PDL‐resistant PWS under previous definitions, may offer additional benefit [80, 102, 103]. Additionally, further research is needed to identify the optimal timing for transitioning to other treatment modalities or to early identify PDL‐resistant PWS patients in order to tailor individual treatment protocols. Non‐invasive detection technology such as optical coherence tomography (OCT) and dermoscopy may help in this regard [104, 105, 106].

#### 4.4.3 Genetic Discoveries in Vascular Anomalies Associated With PWS

PWS manifests cutaneously in several combined vascular anomalies. Advancements in sequencing technology have led to the identification of several associated genes. In 2003, Eerola et al. [107] identified six families exhibiting atypical PWS alongside arteriovenous malformation, arteriovenous fistula, or Parkes Weber syndrome, attributed to RASA1 mutations collectively termed “CM‐AVM”. Shirley et al. [108] in 2013 discovered that Sturge–Weber syndrome and PWS result from a somatic activating mutation in GNAQ. The identification in 2015 of somatic mutations in PIK3CA linked to Klippel‐Trenaunay syndrome places it within the PIK3CA‐related overgrowth spectrum (PROS) [109, 110]. In 2020, a case of Parkes Weber syndrome associated with RASA1 mosaic mutation was reported [111]. Based on these discoveries, the application of clinical genetic testing has promoted diagnostic progress in clinical practice. These findings suggest promising therapeutic targets for future breakthroughs in PWS treatment, emphasizing the necessity for further research into the mechanisms of PWS.

## 5 Conclusion

In this study, we conducted a scientometric analysis to summarize global publication information, research collaborations, and research hotspots in the field of PWS from 2000 to 2023. Furthermore, a meta‐analysis was performed to evaluate the development of PWS treatments. From 2000 to 2023, the publication volume on PWS has remained high but shows stagnation in growth, potentially due to the absence of significant breakthroughs. Although advancements have been made in clearance rates, the improvements are limited. It appears that researchers are prioritizing enhancements to existing energy‐based therapies rather than exploring research directions that could revolutionize current treatment practices.

Future research should focus on several key areas. First, optimizing patient selection, which includes determining the ideal age for treatment initiation, assessing the impact of Fitzpatrick skin types, and refining the definition of PDL‐resistant PWS. Second, defining the threshold for resistance to PDL. Finally, the genetic discoveries in vascular anomalies associated with PWS‐related cutaneous manifestations suggest promising therapeutic targets for future breakthroughs.

In conclusion, our study highlights the current deficiencies in PWS treatment and research, offering valuable insights to guide future investigations and the development of more innovative therapeutic strategies.

## Author Contributions

Z.S., X.C., and R.H. conceptualized the study. Z.S., X.C., and J.B. designed the research. Z.S. and X.C. performed the methodology and formal analysis. R.Z., J.L., and Z.Z. contributed to data curation and visualization. Z.S. and X.C. wrote the original draft. R.H. and J.B. supervised the project, acquired funding, and reviewed and edited the manuscript. All authors have read and approved the final manuscript.

## Ethics Statement

The authors declare that human ethics approval was not required for this study.

## Conflicts of Interest

The authors declare no conflicts of interest.

## Data Availability

The data that support the findings of this study are available from the corresponding author upon reasonable request.
